# From nitrogen addition to productivity: above–belowground mechanisms and nonlinear thresholds in Grasslands

**DOI:** 10.3389/fpls.2025.1719906

**Published:** 2026-01-22

**Authors:** Yujuan Zheng, Xing Zhang, Xiaoxuan Du, Yuchuan Fan, Jie Gao

**Affiliations:** 1 College of Life Sciences, Xinjiang Normal University, Urumqi, Xinjiang, China; 2 College of Ecology and Environment, Xinjiang University, Urumqi, Xinjiang, China; 3 College of Marine and Bioengineering, Yancheng Institute of Technology, Yancheng, Jiangsu, China; 4 Coastal Agriculture Research Institute, Kyungpook National University, Daegu, Republic of Korea; 5 College of Jiyang, Zhejiang A&F University, Zhuji, China; 6 Shaoxing Institute for County-level Common Prosperity Research, Jiyang College of Zhejiang A&F University, Zhuji, China

**Keywords:** context dependency, grassland ecosystems, management framework, net primary productivity, nitrogen input, threshold effects

## Abstract

Grasslands harbor high biodiversity and regulate continental carbon and nitrogen cycling, yet rising anthropogenic nitrogen (N) inputs are reshaping their structure, function, and stability. Synthesizing recent evidence, we show that in N-limited systems moderate N addition tends to raise both ANPP and BNPP by elevating leaf N, optimizing canopy structure, and rebalancing carbon allocation. However, once ecosystem-specific thresholds are exceeded, gains plateau or reverse, coinciding with biodiversity loss, functional-trait homogenization, declines in root-associated mutualists, and soil acidification. N effects are context dependent: thresholds shift lower in dry–hot or semi-arid grasslands and under intense grazing, while soil pH, available phosphorus, and microbial assemblages act as proximal controls that determine whether short-term productivity gains convert into long-term carbon sequestration. We propose a management-ready indicator framework organized along three axes—N dose × water–energy balance × P availability—and paired with field diagnostics (pH, available P, leaf N:P, microbial diversity and key enzyme activities, N_2_O fluxes) to detect early transitions from “moderate” to “excessive” N addition. Priorities include long-term, multifactor experiments and observation–remote sensing–model integration that jointly track plant traits, microbial dynamics, and coupled C–N processes to improve cross-scale prediction and provide actionable guidance for N application and grazing management.

## 1 Introduction

**Figure 1:**
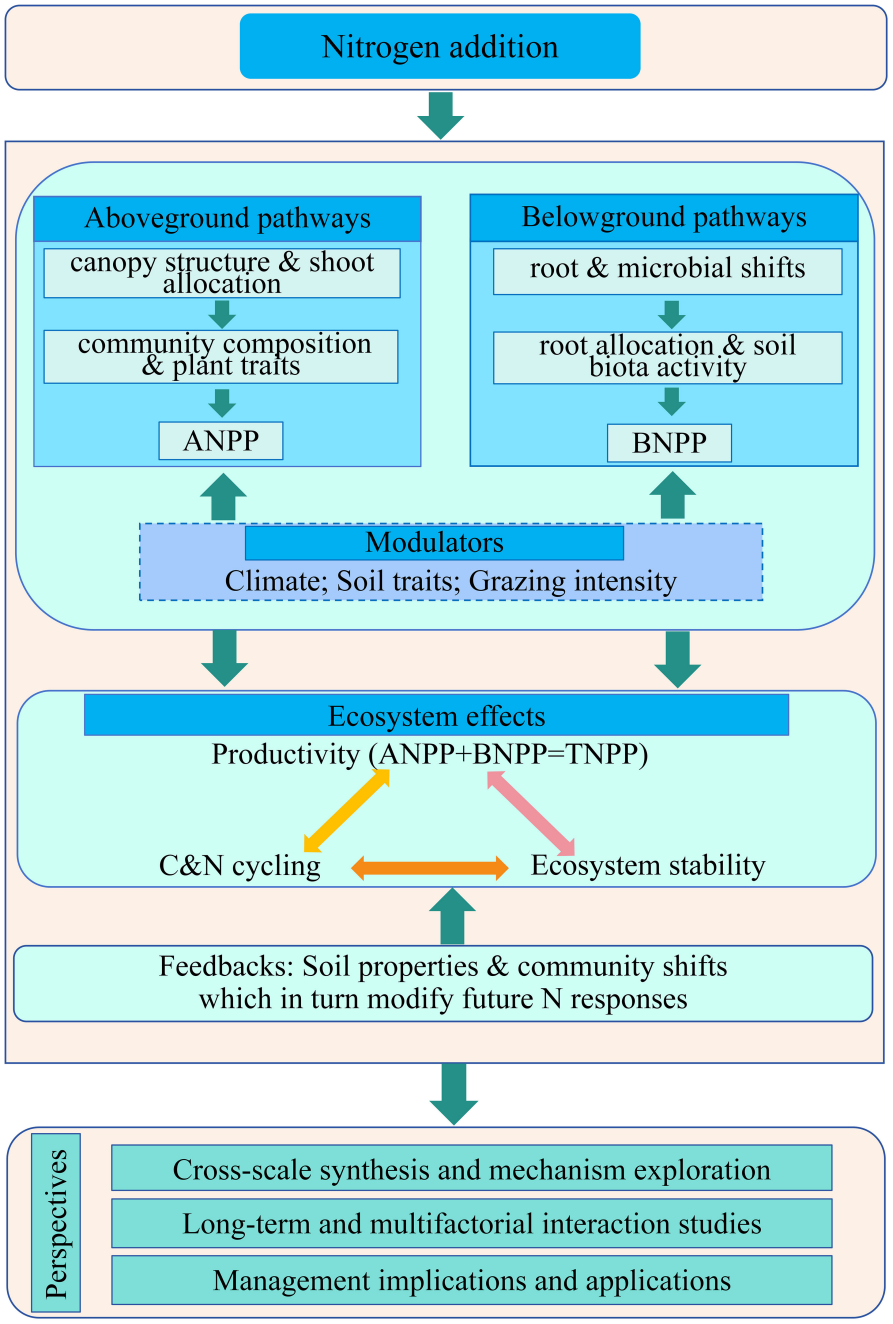
Conceptual framework of nitrogen input–process pathways–ecosystem effects. Nitrogen input influences grassland productivity and stability through two main pathways: the aboveground pathway (canopy structure and aboveground allocation; community composition and plant functional traits), which primarily determines aboveground net primary productivity (ANPP); and the belowground pathway (root allocation and morphology–microbial communities–soil biotic activity), which primarily determines belowground net primary productivity (BNPP). Together, these shape total net primary productivity (TNPP = ANPP + BNPP), and interact with ecosystem stability (interannual stability, resistance–recovery) through carbon–nitrogen cycling processes (mineralization, fixation, loss). External drivers (climate, grazing intensity, soil traits) modulate both pathways and their combined effects in a context-dependent manner. Solid arrows denote primary effect pathways. Abbreviations: ANPP, aboveground net primary productivity; BNPP, belowground net primary productivity; TNPP, total net primary productivity.

**Figure 2:**
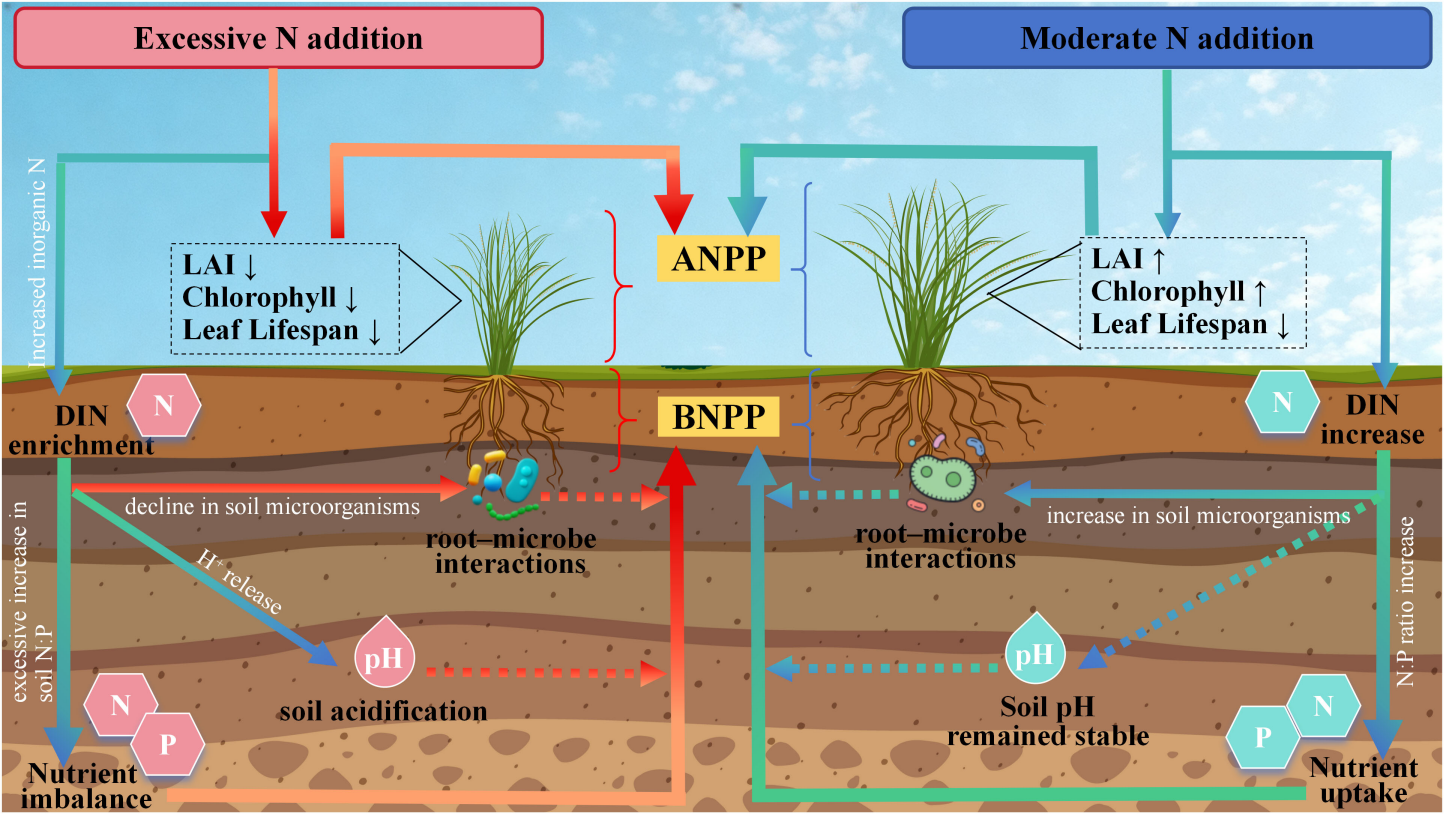
Schematic illustration of contrasting mechanisms under moderate versus excessive nitrogen addition in grasslands. On the right, “moderate nitrogen addition” enhances soil dissolved inorganic nitrogen (DIN) supply while maintaining stoichiometric balance with N–P uptake, stabilizes soil pH, and strengthens root–microbial interactions. These processes increase leaf nitrogen and chlorophyll content, expand leaf area index (LAI), and promote a fast-turnover strategy with shortened leaf lifespan, jointly driving synergistic increases in aboveground net primary productivity (ANPP) and belowground net primary productivity (BNPP). On the left, “excessive nitrogen addition” leads to DIN surplus and N:P stoichiometric imbalance, triggering soil acidification (pH decline) and reduced rhizosphere microbial abundance/interactions. This results in lower LAI and chlorophyll levels, shortened leaf lifespan, and other unfavorable traits, ultimately suppressing both ANPP and BNPP. Solid arrows denote primary causal pathways; dashed arrows indicate indirect or regulatory effects. Teal-colored pathways represent promoting effects, whereas red–orange pathways represent inhibitory or risk effects. All processes are compared with the control without nitrogen addition.

Grasslands cover approximately 40% of the Earth’s terrestrial area and support roughly one-third of the global population (Bengtsson et al., 2019; Du et al., 2021). Against the backdrop of rising global nitrogen (N) inputs, N deposition has shifted from a “background flux” to a key exogenous driver reshaping grassland structure, function, and stability (Stevens et al., 2015; Payne et al., 2017; Reich et al., 2024; Zhu et al., 2025). Nitrogen inputs not only increase the supply of soil dissolved inorganic nitrogen (DIN, e.g., nitrate and ammonium) and thereby alter soil nutrient availability and cycling rates (Bai et al., 2021), but also modulate plant growth and community composition, with cascading effects on ecosystem productivity and interannual stability (Isbell et al., 2013; He et al., 2024). Here, we review decades of research on N addition or deposition impacts on grassland productivity, with a particular focus on the differentiated responses of aboveground net primary productivity (ANPP) and belowground net primary productivity (BNPP), and their underlying mechanisms (**Figures 1**, **2**). We synthesize existing evidence to highlight future research priorities and management strategies, providing a integrative framework for sustainable grassland use and scenario-based predictions.

At the level of direct effects, N inputs generally exhibit intensity-dependent and component-specific impacts on grassland productivity. As a key limiting nutrient, N enrichment typically enhances primary productivity (Bai et al., 2010; Xu et al., 2018), with stronger effects in N-limited grasslands and habitats (Fay et al., 2015). Mechanistically, N addition increases leaf N content and photosynthetic enzyme activity, expands leaf area, and enhances canopy light interception, thereby promoting ANPP (Feng et al., 2023; Liu et al., 2025). Moderate N inputs further improve carbon fixation efficiency and overall productivity (Cao et al., 2025; Tang et al., 2025). However, when N inputs exceed the system’s capacity, marginal gains diminish, plateau, or even decline, often accompanied by accelerated leaf senescence, elevated disease risk, and nutrient imbalance, limiting sustained ANPP enhancement (Xing et al., 2021). In contrast, BNPP responses are more mechanistically coupled and context-dependent, governed by root construction and rhizosphere microbial activity (Han et al., 2023; Sorty et al., 2025). Moderate N addition stimulates root proliferation and water–nutrient uptake (Xu et al., 2017), whereas excessive inputs often shift resource allocation aboveground, reducing root investment and suppressing BNPP (Ma et al., 2023). Moreover, N-induced restructuring of microbial community composition and function influences decomposition and mineralization processes, thereby driving temporal and spatial dynamics of belowground productivity and its stability (Zhang et al., 2018; Liu et al., 2021). Together, these findings highlight the need to simultaneously consider ANPP and BNPP responses, their coupling or decoupling mechanisms (**Figure 2**), and their integrated role in regulating carbon cycling and nutrient dynamics under changing N regimes.

The effects of nitrogen inputs extend beyond mere nutrient supplementation, indirectly shaping productivity patterns through alterations in community structure and functional traits (Zhao et al., 2019; Zhang et al., 2024). Numerous studies have demonstrated that nitrogen addition often reduces species diversity while elevating the dominance of particular species, such as grasses, shifting communities from diversity-driven complementarity toward dominance-driven functional convergence (Song et al., 2011). Concurrently, nitrogen inputs modify root spatial distribution and the rhizosphere environment, influencing microbial composition and function as well as key nutrient fluxes, which ultimately feedback to ecosystem stability and long-term productivity potential (Moreau et al., 2019). Notably, these structural and functional shifts often correspond with N addition thresholds for productivity (**Table 1**). In temperate grasslands, ANPP and BNPP tend to saturate around 7.5–13 and 5–10 g N m^-^² yr^-^¹ (Bai et al., 2010; Wilcots et al., 2022; Yang et al., 2023), respectively. In alpine grasslands, around 10–15 and 2 g N m^-^² yr^-^¹ (Bowman et al., 2006; Zong et al., 2019; Ma et al., 2023). In arid and semi-arid grasslands, around 5–12 and 10 g N m^-^² yr^-^¹ (Li et al., 2011; He et al., 2016; Tang et al., 2017). In grazed grasslands, around 7.5–20 and 10–20 g N m^-^² yr^-^¹ (Gong et al., 2015; Yang et al., 2023; Dong et al., 2024). These effects are strongly modulated by contextual factors including climate (temperature, precipitation) and land use (grazing intensity) (Verburg et al., 2013; Wang et al., 2023). For example, grasslands in arid regions exhibit heightened sensitivity to nitrogen addition and are more prone to reach ecological thresholds (Wang et al., 2017; Shi et al., 2018). Under high grazing pressure, nitrogen inputs may resonate with structural degradation, exacerbating community homogenization and functional risk (Bai et al., 2010). Thus, the interactions between nitrogen addition and environmental factors not only determine the magnitude and direction of productivity responses but also define response windows and threshold positions, providing a fundamental basis for future functional predictions and adaptive management.

**Table 1:** Thresholds represent approximate N addition levels at which productivity responses saturate or shift from positive to neutral or negative.

Ecosystem type/context	TNPP N-addition threshold (g N m^-^² yr^-^¹)	ANPP N-addition threshold (g N m^-^² yr^-^¹)	BNPP N-addition threshold (g N m^-^² yr^-^¹)	References
Temperate grasslands	> 6.7	~ 7.5-13	~ 5-10	Bai et al., 2010; Wang et al., 2019; Wilcots et al., 2022; Cao et al., 2023; Yang et al., 2023;
Alpine grasslands	~ 4-5	~ 10-15	> 2	Bowman et al., 2006; Zong et al., 2019, 2016; Han et al., 2022; Ma et al., 2023
Arid and semi-arid grasslands	> 5	~ 5-12	> 10	Li et al., 2011; He et al., 2016; Chen et al., 2017; Tang et al., 2017;
Grazed grasslands	> 9.5	~ 7.5-10	~ 10-20	Gong et al., 2015; Peng et al., 2020; Yang et al., 2023; Dong et al., 2024
Global grasslands	–	> 15	–	Peng et al., 2020

Values are synthesized from multiple field studies and meta-analyses and thus indicate ranges or lower-bound estimates rather than fixed critical values. Symbols “~”, “>“, and “–” denote approximate ranges, lower-bound thresholds, and unavailable estimates, respectively.

Despite a wealth of empirical evidence (LeBauer and Treseder, 2008; Stevens et al., 2022; Zhou et al., 2017), critical gaps remain. Long-term, multifactorial field data are relatively scarce (Zhang, 2023; Huang et al., 2024), limiting quantitative attribution of the synergistic mechanisms of “nitrogen × climate change × grazing”. Future research should further disentangle the temporal and process-specific responses of ANPP and BNPP, elucidate how nitrogen addition influences belowground carbon pool stability via resource allocation strategies, root structure, and rhizosphere microbial processes (Xing et al., 2021), and integrate functional traits, microbial communities, and ecosystem processes into unified analyses (Bardgett and van der Putten, 2014). Moreover, combining remote sensing (Pettorelli et al., 2018), big data analytics, and ecosystem modeling (Reichstein et al., 2019), will enable cross-scale predictions and scenario extrapolations, ultimately providing a mechanism-based, management-ready framework and diagnostic indicators to support sustainable grassland management and ecological security under global change.

## 2 Direct effects of nitrogen inputs on grassland productivity

### 2.1 Nitrogen limitation and the promotive effects of moderate nitrogen addition

Nitrogen (N) is one of the most common and critical limiting nutrients in grassland ecosystems, playing key roles in the synthesis of proteins, nucleic acids, and chlorophyll, and thereby directly constraining photosynthetic capacity and growth rates (LeBauer and Treseder, 2008). In natural grasslands, soil nitrogen is predominantly organically bound, with plant-available inorganic forms often highly limited (Schimel and Bennett, 2004), making productivity largely dependent on external nitrogen inputs (Harpole et al., 2016). As nitrogen deposition or fertilization increases, the availability of DIN (particularly ammonium and nitrate) rises, alleviating N limitation and promoting leaf expansion (Pettorelli et al., 2018), photosynthetic efficiency, and aboveground biomass accumulation (Reich et al., 2006; Lan and Bai, 2012).

Field manipulations across multiple sites and meta-analyses consistently show that moderate nitrogen addition reproducibly enhances productivity (Bai et al., 2010; You et al., 2017) (**Table 1**). At the global scale, nitrogen inputs have been estimated to increase aboveground net primary productivity (ANPP) by ~29% on average (LeBauer and Treseder, 2008), with another synthesis of 304 field experiments reporting an approximate 42% increase in aboveground biomass (Xia and Wan, 2008). The underlying physiological–ecological pathway is clear. Nitrogen enhances leaf photosynthetic capacity and related enzymatic activity, increases chlorophyll content, and expands leaf area index (LAI), translating leaf-level gains along the leaf economics spectrum to canopy-scale light capture and carbon assimilation (Adams et al., 2016; Khan et al., 2022). At the community level, these physiological gains are further amplified through interspecific resource partitioning and optimized canopy structure, with dominant species showing relatively greater responses and collectively driving aboveground biomass accumulation (Jia et al., 2022; Shen et al., 2022). Overall, this “bottom-up” promotive effect is particularly pronounced in temperate and alpine grasslands, highlighting nitrogen as a primary limiting factor with a pervasive and critical role in regulating grassland productivity (Song et al., 2011; Zhang et al., 2022). The conceptual framework illustrating how nitrogen inputs affect grassland productivity and stability is summarized in **Figure 1**, showing the pathways through which nitrogen influences above-and belowground processes, ultimately shaping total net primary productivity (TNPP) and ecosystem stability.

### 2.2 Responses of ANPP to nitrogen inputs and threshold mechanisms

The positive effects of nitrogen (N) on aboveground net primary productivity (ANPP) are well-established. However, they arise from a multilevel, interconnected chain of mechanisms. First, N enhances chlorophyll content and the activity of photosynthesis-related enzymes, thereby increasing maximum assimilation rates per unit leaf area and improving light-use efficiency (Liang et al., 2020). Second, by stimulating increases in leaf area index (LAI) and canopy height, N promotes light interception and distribution at the community level, accelerating carbon inputs and driving aboveground biomass accumulation (Guo et al., 2016; Yuan et al., 2025). Third, N improves internal nutrient supply, enabling plants to sustain higher leaf production and growth rates, albeit often at the cost of shortened leaf lifespan (Wright et al., 2004). As illustrated in **Figure 2**, these processes are temporally sequential and spatially integrated—from leaf-level expansion to canopy development—allowing the “N–photosynthesis–structure” positive feedback to scale up into stable gains in ANPP.

However, this promotion is not monotonic or linear (**Table 1**). Increasing evidence suggests that the ANPP–N input relationship follows threshold or saturation dynamics (Tian et al., 2016). Once inputs exceed the system’s carrying capacity, excessive N induces nutrient imbalances (e.g., relative scarcity of K, Mg, and P), increases toxicity risks, and elevates pathogen susceptibility, collectively suppressing productivity (Ren et al., 2025). For instance, long-term fertilization in European temperate grasslands has shown that excessive N elevates leaf N in certain grasses into stress-inducing ranges and increases disease incidence, ultimately constraining community-level productivity (Bobbink et al., 2010). Similarly, in North American prairies, high N deposition alters leaf nutrient stoichiometry and suppresses fine root growth, indirectly limiting ANPP by weakening water and mineral supply from belowground (Stevens et al., 2004).

In summary, ANPP responses depend on the balance between N dosage and environmental carrying capacity. Within an optimal range, N markedly enhances photosynthesis and community-level light utilization (Ren et al., 2024). Beyond the threshold, processes such as physiological stress, soil chemical deterioration, and structural homogenization offset or even reverse positive effects (de Vries, 2021). Thus, evaluating the impacts of N inputs on ANPP requires explicit consideration of nonlinear thresholds and long-term stability, avoiding simplistic linear extrapolation (Meng et al., 2021).

### 2.3 Responses of BNPP to nitrogen inputs and allocation trade-offs

Belowground net primary productivity (BNPP) integrates root growth and rhizosphere microbial activity, underpinning critical functions such as water and nutrient uptake, soil organic carbon (SOC) accumulation, and carbon pool stability (Jackson et al., 1996). BNPP responses to nitrogen (N) inputs are more context-dependent and bidirectional compared with ANPP (Yuan and Chen, 2012). Under moderate N inputs, root growth is stimulated, leading to increases in root length and biomass, which improve water and mineral interception efficiency and thereby enhance belowground biomass accumulation (Fan et al., 2009). At the same time, N enrichment can restructure rhizosphere microbial communities and functional profiles, elevating metabolic activity and nutrient cycling capacity, which indirectly supports BNPP (Yue et al., 2016). For example, in the Inner Mongolian steppe, low-to-moderate N fertilization significantly increased root length and biomass, thereby enhancing belowground carbon inputs (Lü et al., 2013).

In contrast, under excessive N inputs, plants in nutrient-rich conditions often reallocate more carbon aboveground to strengthen light competition, reducing investment in root systems and causing BNPP decline (Cao et al., 2023). Chronic fertilization may also increase root respiration and carbon consumption, undermining the long-term stability of belowground carbon pools (Keller et al., 2023). Collectively, these findings indicate that BNPP exhibits clear threshold responses to N: beyond the ecological carrying capacity, negative effects rapidly emerge (**Table 1**).

Importantly, the differential responses of ANPP and BNPP to N inputs reshape above–belowground carbon allocation, with consequences for SOC accumulation and the long-term carbon sequestration capacity of grassland ecosystems (Ma et al., 2023). These allocation shifts are spatially heterogeneous. In mesic grasslands, water and energy availability can synergistically amplify BNPP responses, whereas in arid systems, water limitation constrains N benefits and can even reverse them (Bai et al., 2015; Xu et al., 2024). Thus, understanding the threshold- and context-dependence of BNPP is essential for predicting global grassland C–N dynamics and informing adaptive management strategies (Yang et al., 2023).

## 3 Indirect effects of nitrogen inputs on grassland ecosystem structure and function

### 3.1 Species composition and functional trait reorganization: from diversity loss to competitive shifts

As shown in **Figure 1**, nitrogen (N) inputs not only increase soil N availability but also amplify asymmetries in resource competition, thereby reshaping dominance hierarchies and functional group proportions within grassland communities (Suding et al., 2005). The underlying mechanism lies in the strengthened competitive ability of a few dominant species under high-N conditions (Hautier et al., 2009). These species achieve faster canopy closure and occupy the upper light environment and shallow soil resources, thereby squeezing the niche space of other taxa (Isbell et al., 2013). Biodiversity loss entails more than a simple reduction in species richness—it directly undermines complementarity and stability mechanisms (Hooper et al., 2005). For instance, the decline of N-fixing species or deep-rooted forbs weakens the system’s capacity for biological N inputs and deep-soil water and nutrient replenishment, increasing ecosystem sensitivity to external N supply and interannual fluctuations (Fornara and Tilman, 2009). This structural reorganization helps explain the widespread pattern of “short-term productivity gains but long-term resilience decline” under N enrichment. Initial ANPP increases are followed by losses of resistance and recovery capacity as communities become more homogeneous and functionally convergent, making productivity more vulnerable to climatic extremes or disturbances (Isbell et al., 2013).

At the same time, N inputs reshape key functional traits by increasing leaf nutrient concentrations but shortening leaf lifespans, driving a shift toward “fast-turnover, high-assimilation, low-persistence” strategies (Reich et al., 1997; Liang et al., 2020). While these strategies boost photosynthesis and biomass accumulation in the short term (Sun et al., 2022), they also heighten dependence on water and phosphorus. The associated imbalances in C:N:P stoichiometry constrain nutrient recycling and root-based resupply, thereby indirectly reducing long-term ecosystem productivity (Sardans et al., 2012). Collectively, N inputs influence grassland productivity indirectly through a coupled pathway linking community composition, functional traits, and ecological processes.

### 3.2 Rhizosphere microbial assembly and process reallocation: reshaping nitrogen fluxes and C–N coupling

Plant roots and rhizosphere microbes together form the central hub of nutrient cycling in grasslands, with their assembly rules directly influencing water and mineral acquisition as well as nitrogen transformation and regeneration (Zhou and Ning, 2017). Nitrogen inputs alter the nitrogen supply and soil pH background, thereby reshaping microbial community diversity, composition, and function (Leff et al., 2015). Elevated nitrogen generally suppresses diazotrophs and reduces the system’s self-nitrogen-fixation capacity (Wang et al., 2024), while altering the relative activity of nitrifiers and denitrifiers. This reconfigures the distribution of inorganic nitrogen forms and the retention–loss balance, which feeds back to plant nitrogen use efficiency and growth (Ren et al., 2024) (**Figure 2**).

Microbial functional reorganization also strongly affects carbon cycling. Under conditions of nitrogen enrichment, decomposition enzyme spectra shift toward faster turnover pathways, weakening soil organic matter protection and accelerating carbon cycling, thereby diminishing positive feedbacks to BNPP (Riggs et al., 2015; Riggs and Hobbie, 2016). Long-term or high-intensity nitrogen inputs may reduce microbial diversity and enzyme activity, lowering decomposition and mineralization efficiency (Wang et al., 2019; Hou et al., 2021), thus limiting nutrient availability for plants and creating a paradox of “nitrogen enrichment but low efficiency.”

In sum, nitrogen addition regulates rhizosphere microbial assembly and the relative importance of key functional groups (N-fixers, nitrifiers, denitrifiers), thereby altering nitrogen cycling rates and pathways. Through C–N interactions, this indirectly shapes grassland productivity (Ren et al., 2024). This complexity underscores the need to integrate plant–microbe interactions into evaluation frameworks to detect early-warning signals of “apparent productivity gains but actual instability” (Zhou et al., 2021).

### 3.3 Integrated ecosystem responses: nonlinear trade-offs in productivity, stability, and multifunctionality

From a multifunctionality perspective, nitrogen inputs alter productivity, stability, and regulatory services via interconnected pathways linking communities, microbes, and soil chemistry (Bobbink et al., 2010) (**Figure 2**). In the short term, biomass gains and canopy closure of dominant species typically enhance carbon fixation (Suding et al., 2005). This “rise-and-fall” trajectory aligns with shifts in species composition and trait strategies described above. On the microbial side, functional degradation further decreases nutrient cycling efficiency, manifested as downregulation of enzyme activity and declines in mineralization and regeneration fluxes, which restrict nitrogen and phosphorus availability to plants (Riggs et al., 2015; Hou et al., 2021). Microbial functional degradation continues to reduce nutrient cycling efficiency, limiting nitrogen and phosphorus availability to plants (Stevens et al., 2004; Guo et al., 2010; Wei et al., 2013; Tian and Niu, 2015).

Overall, ecosystem responses to nitrogen inputs exhibit strong nonlinearity and context dependence. Short-term productivity gains do not guarantee long-term stability. Identifying and managing thresholds and early-warning signalsis crucial to prevent a shift from “enhancement to degradation”.

### 3.4 Soil chemistry and stoichiometric gatekeeping: acidification, phosphorus limitation, and physical protection

Nitrogen inputs profoundly affect the “upper and lower bounds” of productivity responses by modifying soil chemistry. Long-term or high-intensity inputs cause acidification (pH decline, base cation loss), mobilizing toxic ions such as Al³^+^ that directly suppress root elongation and nutrient uptake (Guo et al., 2010; Tian and Niu, 2015). Acidification also alters microbial community composition and enzyme systems, shifting decomposition pathways toward faster turnover, weakening the physical–chemical protection of soil organic matter, and limiting the translation of productivity gains into carbon sequestration (Zhao et al., 2020).

Phosphorus (P) limitation is another key constraint in nitrogen-enriched systems. When nitrogen increases without matching phosphorus supply, plant P demand rises but effective P availability often declines due to acidification and mineral sorption, resulting in C:N:P imbalance and limiting sustained growth of leaves and roots (Reich et al., 2006; Sardans et al., 2012; Lu et al., 2014). In such cases, even “moderate” nitrogen doses can trigger productivity plateaus or declines, particularly evident in BNPP reductions (Lu et al., 2011; Yue et al., 2016).

Soil texture and mineralogy further determine the strength of organic matter and nitrogen stabilization. Fine-textured soils with high CEC and Fe–Al oxides can form stable organo-mineral complexes, delaying nitrogen saturation and productivity plateaus. In contrast, sandy soils and low-SOM backgrounds are more prone to leaching and early responses (Hassink, 1992; Zhao et al., 2020). Management strategies such as phosphorus supplementation, pH buffering, organic amendments, or nitrogen form adjustment can lower chemical thresholds, extend the “moderate nitrogen window,” and enhance long-term carbon sequestration (Cao et al., 2025; Tang et al., 2025).

### 3.5 Climatic and grazing modulation: shifting thresholds left or right

Nitrogen effects are tightly coupled with water and energy conditions. Under hot–dry climates (low MAP, high temperature and high evapotranspiration), water limitation becomes primary, accelerating the decline of nitrogen marginal returns and shifting the “moderate-to-excessive” threshold leftward. In contrast, in wetter years or regions, the effective nitrogen window extends (Fay et al., 2015; DeMalach et al., 2017). Climatic variability also influences greenhouse gas fluxes. Higher precipitation can amplify the sensitivity of N_2_O emissions to nitrogen inputs (Du et al., 2021; Diao et al., 2025), increasing the environmental costs of productivity gains.

Grazing modifies above- and belowground inputs and spatial heterogeneity, acting as either an accelerator or buffer under nitrogen enrichment. Moderate grazing can reduce dominance monopolization, maintain microsite heterogeneity, and widen the safe space for moderate nitrogen. In contrast, heavy grazing under nitrogen enrichment accelerates homogenization and degradation, narrowing the effective window (Milchunas and Lauenroth, 1993; Zhang et al., 2017; Zhou et al., 2017; Maestre et al., 2022; Sheng et al., 2023). Moreover, grazing–fertilization interactions reshape microbial metabolism and C–N fluxes, altering the net response of systems to nitrogen, particularly in semiarid grasslands (Wang et al., 2017; Shi et al., 2018; Qi et al., 2023; Wang et al., 2023). Thus, nitrogen dose, climate variability, and grazing intensity should be jointly considered as interactive drivers. Dynamic regulation based on thresholds and early signals (e.g., microbial diversity, N_2_O fluxes) is essential for sustainable grassland management (**Figure 1**).

## 4 Interactions of nitrogen input with other environmental factors

### 4.1 Synergistic effects of nitrogen input and climate conditions on grassland productivity

The effects of nitrogen are strongly modulated by the water–energy context, as precipitation and temperature reshape the “effective window” of nitrogen through regulating soil moisture availability, microbial activity, and mineralization rates (LeBauer and Treseder, 2008; Diao et al., 2025). In humid and thermally favorable regions, nitrogen addition alleviates N limitation and interacts with enhanced mineralization and assimilation to elevate both ANPP and BNPP (Guo et al., 2016). Long-term observations indicate that subtropical grasslands (Rodríguez Palma et al., 2024) and temperate meadows display pronounced biomass increases under moderate N input, with warming further reinforcing this positive effect by accelerating decomposition and nutrient cycling (Bai et al., 2013; Henry et al., 2015; Zhang et al., 2015). In contrast, water scarcity in arid and semiarid systems raises the entry threshold for N use, with low soil moisture and suppressed enzymatic activity limiting N transformation and growth response (Chen et al., 2019; Bondaruk et al., 2025) (**Table 1**). Warming and altered precipitation regimes further enhance the context dependence of nitrogen effects, shifting leaching, volatilization, and runoff pathways, and reshaping C–N coupling (DeMalach et al., 2017). Regional differences are evident: alpine meadows on the Qinghai–Tibetan Plateau exhibit stronger microbial and productivity responses to N addition than drought-prone temperate steppes (Bai et al., 2004; Han et al., 2022).

### 4.2 Interactive effects of nitrogen input and grazing intensity

Grazing modifies above–belowground inputs, spatial heterogeneity, and soil physical structure, thereby interacting with nitrogen input (Maestre et al., 2022). Moderate grazing constrains dominant species expansion, buffers N-induced homogenization, and maintains functional diversity and microsite heterogeneity, stabilizing or amplifying the positive effects of nitrogen (Milchunas and Lauenroth, 1993; Sheng et al., 2023). Empirical evidence shows that light grazing plus moderate N enhances ANPP and BNPP concurrently while sustaining microbial diversity and activity (Wang et al., 2021; Usman et al., 2025). In contrast, heavy grazing combined with high N acts as a degradation accelerator, reducing vegetation cover, soil porosity, and community stability, while increasing susceptibility to drought and extreme conditions (Hautier et al., 2014; Lai and Kumar, 2020). Livestock excreta further alter soil chemistry and microbial assembly, producing short-term boosts but long-term risks of salinization and imbalance (Zhang et al., 2017; Qi et al., 2023; Wang et al., 2023). Joint management of N load and grazing intensity is thus critical to avoid threshold crossings and degradation trajectories (Ren et al., 2019).

### 4.3 Soil properties as modulators of nitrogen effects

Soil physicochemical characteristics critically determine N transformation, retention, and bioavailability (Lu et al., 2014). Soil pH is a primary factor. N input often exacerbates acidification, leading to base cation loss, metal mobilization, and reduced microbial diversity and nitrification efficiency (Keeler et al., 2009; Guo et al., 2010) (**Figure 2**). Coupled pH decline and phosphorus depletion create hidden thresholds where nominally moderate N becomes effectively excessive (Sardans et al., 2012). Organic matter enhances buffering capacity and nutrient retention, sustaining microbial function and productivity (Six et al., 2006), whereas sandy, low-carbon soils are prone to leaching and volatilization, increasing degradation risk (Zhao et al., 2020). Soil texture mediates response thresholds, with fine-textured soils exhibiting higher N use efficiency, and coarse-textured soils showing earlier saturation (Hassink, 1992; Wang et al., 2022).

### 4.4 Nitrogen form and stoichiometric matching

The form of nitrogen (NH_4_^+^, NO_3_^-^, or organic) and its stoichiometric compatibility with phosphorus and potassium jointly define the effective boundary between moderate and excessive inputs. Single N addition under P deficiency frequently triggers C:N:P imbalance, constraining growth and accelerating plateau onset (Sardans et al., 2012; Lu et al., 2014). Long-term co-application of P or organic amendments enhances P availability, and stabilizes organo-mineral complexes, extending the “moderate N window” (Cao et al., 2025; Tang et al., 2025). N form further influences volatilization, leaching, and rhizosphere micro-pH, underscoring the need for integrated management of “dose × form × stoichiometry” (Keeler et al., 2009; Zhao et al., 2020).

### 4.5 Temporal and spatial scales, extreme events, and thresholds

Nitrogen effects exhibit marked temporal nonlinearity. Short-term stimulation is followed by mid- to long-term plateaus or declines, often coinciding with acidification, P limitation, and community convergence (Tian et al., 2016; Meng et al., 2021) (**Table 1**). Extreme events such as droughts, heatwaves, and floods rapidly compress the moderate N window, forcing threshold transitions into low-function states with ANPP–BNPP decoupling and stability loss (DeMalach et al., 2017; Wu et al., 2020). Spatially, water–energy regimes, soil traits, and grazing intensity jointly shape regional thresholds. Warming plus moderate N may unlock productivity potential in cold–wet alpine grasslands, while arid temperate grasslands reach saturation earlier (Bai et al., 2004; Han et al., 2022; Bondaruk et al., 2025). Zone-specific threshold management is thus recommended, using early-warning indicators such as pH decline, leaf N:P ratios, soil organic matter, N_2_O fluxes, and microbial diversity (Du et al., 2021; Wang et al., 2021; Diao et al., 2025).

## 5 Management implications, diagnostic indicators, and predictive frameworks

### 5.1 A conceptual response framework: N dosage × water–energy × P availability

Synthesizing evidence from Sections 2–4, we propose a conceptual response framework defined by nitrogen dosage (moderate–excessive) as the primary axis, modulated by water–energy background (precipitation and temperature) and P availability and soil chemical gating as two key axes. This framework explains why identical nitrogen inputs yield contrasting outcomes across regions. In humid, near-neutral, organic-rich soils, moderate N additions are more likely to translate into stable ANPP and BNPP gains, whereas in dry–hot, acidic, low-P settings, even nominally “moderate” N often leads to premature plateauing or decline due to stoichiometric imbalance and acidification (Sardans et al., 2012; Tian and Niu, 2015; Fay et al., 2015; DeMalach et al., 2017).

Here, we present it as a conceptual proposal to guide future research and explore potential management implications under varying conditions. For example, prioritize yield-enhancing N application where water is not limiting and P is adequate. But in arid and semi-arid or low-P and acid-prone soils, nitrogen should be coupled with P supplementation, pH buffering, or organic amendments. Otherwise, marginal yield benefits decline rapidly while stability costs accumulate (Guo et al., 2010; Tang et al., 2025; Cao et al., 2025). For grasslands aimed at long-term carbon sequestration or stability (e.g., conservation areas, ecological barriers), nitrogen strategies should shift from maximizing annual yield to prolonging the “moderate window” and minimizing threshold exceedance risk (Wu et al., 2020; Meng et al., 2021).

### 5.2 Field-applicable diagnostic indicators and threshold zones

Based on existing experiments and meta-analyses, we recommend a set of early-warning and process-diagnostic indicators. These include soil pH, available P, leaf N, LAI and canopy height, microbial diversity and enzyme activity, and N_2_O fluxes. Practically, a “latent threshold warning zone” can be defined. Key signals include pH decline ≥ 0.1–0.2 within 3–5 years, community-weighted mean leaf N > 16–20, and progressive declines in available P coupled with rising LAI and saturated Amax, all indicators are signaling an approach toward the productivity plateau–stability decline threshold (Hautier et al., 2009; Sardans et al., 2012; Tian and Niu, 2015; Liang et al., 2020). In this zone, strategies such as P co-application, liming and biochar buffering, or organic amendments should be prioritized (Cao et al., 2025; Tang et al., 2025).

On the microbial side, diversity loss, imbalance among functional groups (N-fixation, nitrification, denitrification), and shifts toward fast-turnover enzyme profiles often parallel BNPP weakening (Leff et al., 2015; Riggs et al., 2015; Riggs and Hobbie, 2016; Wang et al., 2019; Hou et al., 2021). In grazed systems, monitoring conductivity or salinity and N_2_O fluxes in dung or urine hotspots provides critical signals of N × grazing interactions driving secondary soil stress and spillover risks (Du et al., 2021; Qi et al., 2023; Diao et al., 2025).

### 5.3 Integrated optimization of fertilization and grazing

Given the combined constraints of acidification, P gating, and community convergence, coordinated optimization across dosage, N form, nutrient co-application, and grazing is essential. Dosage management should follow a “moderation-centered, site-specific quota” principle. In practice, this means higher moderate thresholds in humid, neutral soils, and stricter down-regulation in dry, acid-prone, P-limited systems with mandatory P supplementation and pH buffering (Guo et al., 2010; Sardans et al., 2012). In terms of N form, in acid-sensitive soils, strategies should minimize losses via nitrification–denitrification and ammonia volatilization by adopting organic N, controlled-release fertilizers, or surface-covering practices (Keeler et al., 2009; Zhao et al., 2020).

Moderate grazing can sustain microsite heterogeneity and functional diversity, expanding the “moderate N window,” whereas heavy grazing under high N amplifies compaction, homogenization, and instability (Lai and Kumar, 2020; Sandoval-Calderon et al., 2025).

### 5.4 Monitoring–model integration: from site diagnosis to regional prediction

Scaling site-level diagnostics to regional prediction requires integration of field monitoring, remote sensing, and process-informed and data-driven modeling. Remote sensing provides multi-scale indicators such as LAI, NDVI, canopy structure, and water status for spatially explicit tracking of nitrogen windows and temporal thresholds (Pettorelli et al., 2018). Modeling efforts should incorporate microbial assembly, acidification–P gating, community convergence, and grazing–excreta inputs into land-surface models or apply deep learning and interpretable machine learning for data assimilation, allowing explicit representation of nonlinearities, thresholds, and indirect pathways to enhance predictability (Hooper et al., 2005; Leff et al., 2015; Reichstein et al., 2019).

We further recommend standardized nitrogen reporting protocols: annual dosage (kg N·ha^-^¹·yr^-^¹), N form, application timing, soil pH, available P, organic matter, grazing intensity and stocking rates, and N_2_O fluxes or estimates. Such reporting will improve cross-study comparability and reduce uncertainty in meta-analyses and regional extrapolations (LeBauer and Treseder, 2008; Xia and Wan, 2008; Zhu et al., 2025).

### 5.5 Research design and knowledge gaps: from “single-factor short-term” to “multi-factor long-term”

Current evidence remains constrained by short-duration, single-factor, site-specific experiments, limiting detection of threshold responses under climate–soil–grazing interactions (Zhang et al., 2023; Huang et al., 2024). Future designs should incorporate long-term multi-factor factorial or semi-factorial experiments combining N dosage, water (irrigation or exclusion), temperature (warming), grazing intensity, and P, organic amendments, jointly measuring ANPP and BNPP, community traits, microbial activity, soil chemistry and mineralogy, and greenhouse gas fluxes to identify causal pathways and threshold zones (Bai et al., 2013; DeMalach et al., 2017; Maestre et al., 2022).

Belowground processes remain a key blind spot: direct BNPP measurements, fine-root turnover and trait syndromes, microbial carbon use efficiency and functional redundancy, and archaeal, bacterial and fungal partitioning under combined N–drought–grazing stress all require long-term quantification (Jackson et al., 1996; Yuan and Chen, 2012; Xu et al., 2025). Furthermore, stability and multifunctionality should be elevated alongside productivity as core decision metrics, clarifying trade-offs among yield, stability, and environmental spillover (Wu et al., 2020).

## 6 Conclusion

Synthesizing existing evidence, the effects of nitrogen input on grassland productivity are strongly intensity- and context-dependent. Under conditions where water and phosphorus (P) are not limiting and soil acidity remains controllable, moderate N additions enhance leaf N content and photosynthetic capacity, expand canopy structure, and optimize resource acquisition—stabilizing ANPP gains while enabling BNPP to increase in parallel without compromising root investment. Once inputs exceed ecosystem carrying thresholds, however, indirect effects such as acidification, P gating, community convergence, and rhizosphere microbial functional decline accumulate rapidly, shifting benefits into plateaus or declines, accompanied by reduced stability and elevated spillover risks such as N_2_O emissions. Consequently, the same nitrogen addition may lead to divergent ecological trajectories depending on water–energy context, soil traits, and grazing use, with thresholds shifting according to environmental conditions.

Mechanistically, nitrogen reshapes ecosystems through a “composition–trait–process” triad. At the community level, by reinforcing dominance and reducing diversity and complementarity. At the trait level, by shifting strategies toward “fast turnover—high assimilation, low persistence”. At the process level, by altering microbial assembly and nitrogen fluxes, which, in interaction with acidification and P constraints, ultimately determine above–belowground carbon allocation and long-term sequestration potential.

From a management perspective, strategies should move away from the linear pursuit of maximum annual yield toward a risk-sensitive framework that emphasizes extending the “moderate window” and preventing threshold crossings. This requires situating nitrogen dosage within a coordinate system of N × water–energy × available P, complemented by form management and co-application (P amendments, organic inputs, pH buffering), alongside moderate grazing to maintain spatial heterogeneity and microbial function.

For field diagnostics, we recommend early-warning indicators including soil pH, available P, leaf N:P ratios, LAI and canopy height, microbial diversity and key enzyme activities, and N_2_O fluxes. Deploying these indicators for zone-specific thresholds and climate-year adjustments can reduce the likelihood of degradation transitions under “high N × heavy grazing × dry-hot year” scenarios.

Future research must prioritize long-term, multi-factor experiments and the integration of models, remote sensing, and monitoring to explicitly capture nonlinearities and causal pathways. Closing critical knowledge gaps requires quantification of BNPP, fine-root turnover, microbial carbon use efficiency, and belowground functional redundancies. Moreover, stability and multifunctionality should be elevated alongside productivity as explicit management targets.

In sum, embedding mechanistic thresholds and operational diagnostics into decision-making offers a feasible pathway to simultaneously secure productivity, stability, and low spillover in grasslands under global change and chronic nitrogen enrichment.
